# Nanomaterial ZnO Synthesis and Its Photocatalytic Applications: A Review

**DOI:** 10.3390/nano15090682

**Published:** 2025-04-30

**Authors:** Chunxiang Zhu, Xihui Wang

**Affiliations:** 1 Institute of Materials Science and Engineering, University of Connecticut, Storrs, CT 06268, USA; 2 Department of Communication, University of Connecticut, Storrs, CT 06268, USA; xihui.wang@uconn.edu

**Keywords:** ZnO nanomaterials, photocatalysis, dye photo degradation, water treatment, H_2_ generation, CO_2_ reduction

## Abstract

Zinc oxide (ZnO), a cheap, abundant, biocompatible, and wide band gap semiconductor material with easy tunable morphologies and properties, makes it one of the mostly studied metal oxides in the area of materials science, physics, chemistry, biochemistry, and solid-state electronics. Its versatility, easy bandgap engineering with transitional and rare earth metals, as well as the diverse nanomorphology empower ZnO as a promising photocatalyst. The use of ZnO as a functional material is attracting increased attention both for academia and industry, especially under the current energy paradigm shift toward clean and renewable sources. Extensive work has been performed in recent years using ZnO as an active component for different photocatalytic applications. Therefore, a thorough and timely review of the process is necessary. The aim of this review is to provide a general summary of the current state of ZnO nanostructures, synthesis strategies, and modification approaches, with the main application focus on varied photocatalysis applications, serving as an introduction, a reference, and an inspiration for future research.

## 1. Introduction

**Figure 1:**
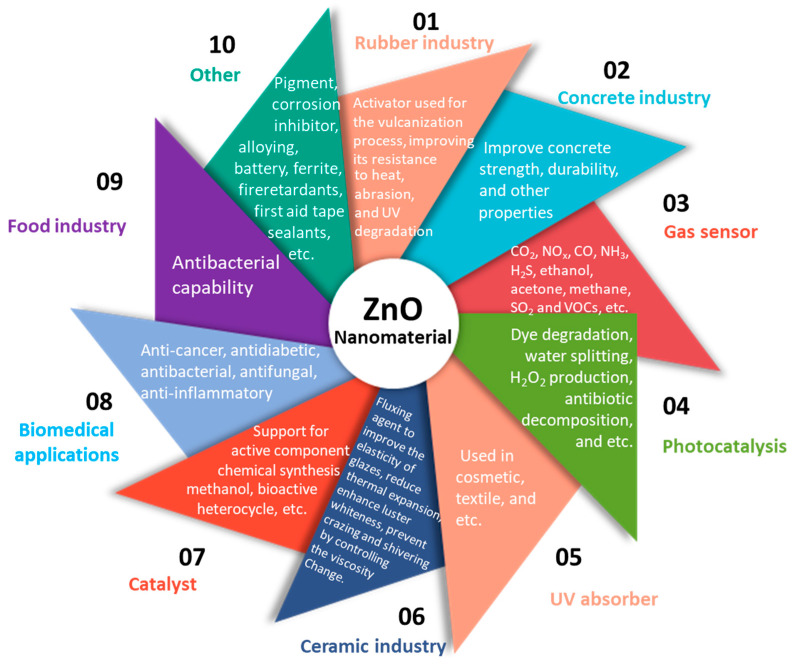
Different applications of ZnO based nanomaterials and brief description of the application.

Zinc oxide (ZnO) is a low-cost, abundant, and safe material that has wide applications in many important areas of our daily lives. For instance, in the rubber industry, ZnO is used as a vulcanization accelerator activator for tire production, consuming between 50% and 60% of ZnO usage in general. It is also used to modify the ceramic properties in concrete manufacturing, including mechanical properties, workability, and appearance. In skin ointments and sunblock creams, ZnO is used to absorb UV light, offering a protective layer. In the food industry, ZnO is used as a source of zinc. As one of the most important semiconductors, ZnO has been extensively studied for gas sensors [1,2,3,4], solar cells [5], transducers [6,7], photodetectors [8], transistors [9], catalysts [10,11,12], and so on. The detailed ZnO nanomaterial applications are summarized and shown in Figure 1.

ZnO is the second most common metal oxide in the Earth’s crust, following iron [13]. Naturally occurring as the mineral zincite, ZnO typically appears as a white powder with very low solubility in water. Its use and scientific exploration date back thousands of years. Historical records show that as early as 2000 B.C., ZnO was applied in ointments to treat various skin ailments in ancient Egypt and later in Roman society. During the Middle Ages, ZnO ore was used in brass manufacturing in Europe and was especially significant in regions of central and eastern Asia. By the 18th century, ZnO became widely used as a pigment, gaining popularity in watercolor art during the 19th century. The introduction of the French process further expanded its use in oil painting [13]. ZnO has been scientifically studied since at least 1912 [14], and research on its semiconductor properties has been conducted since the 1950s and 1970s. In 1960, ZnO was discovered to have piezoelectric properties and was used as a thin electron layer in surface acoustic wave devices. ZnO has many unique chemical and physical properties, such as high chemical stability, high electrochemical coupling coefficient, broad range of radiation absorption, and high photostability, which make it one of the key metal oxides for technological material, and it has wide applications. ZnO also possesses a range of favorable optical and electrical characteristics, including excellent transparency, high electron mobility, a large exciton binding energy (60 meV), and a wide band gap (3.37 eV). It can exhibit strong luminescence at room temperature, as well as notable thermal and mechanical stability. Additionally, ZnO has a broad absorption spectrum and high resistance to photodegradation, making it a highly versatile engineering material. Due to these unique properties, ZnO is considered a promising candidate for use in electronics, optoelectronics, and laser technologies. More importantly, the physical and chemical behavior of ZnO nanoparticles can be effectively tailored by adjusting their nanostructure through the use of different synthesis methods and precursor materials [13].

**Figure 2:**
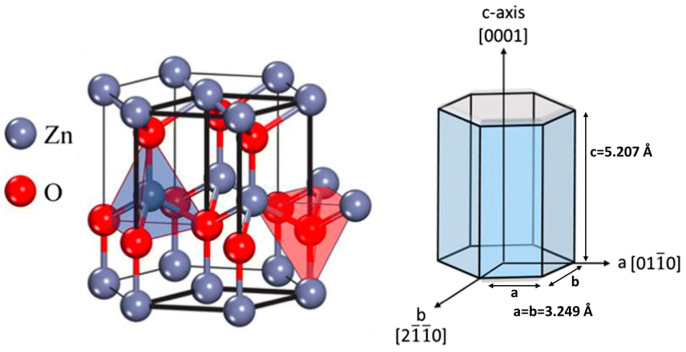
Wurtzite crystal structure of ZnO with hexagonal unit cell (**Left**: unit cell marked as red or blue color) and different crystallographic facets (**Right**). Adopted from ref. [15].

ZnO has three different crystal structures, wurtzite, rock-salt, and zinc-blende; in ambient conditions, wurtzite structure is the most thermodynamically stable phase. As shown in Figure 2, ZnO has a hexagonal wurtzite crystal structure with a, b, and c lattice parameters, where a = b = 0.3296 nm and c = 0.52065 nm. In the hexagonal wurtzite structure, O^2−^ ions occupy the hexagonal close packed (hcp) array of lattice sites while Zn^2+^ ions place themselves in alternate tetrahedral holes. The ZnO primitive unit cell (shown as a thicker black line in Figure 2) has two formula units, either one Zn ion surrounded by four O ions in a tetrahedral coordination or vice versa [15]. ZnO exhibits partially ionic bonding and lacks a center of inversion, which gives rise to its piezoelectric properties. The non-centrosymmetric tetrahedral coordination within its crystal structure leads to crystallographic polarity, a crucial factor influencing both its crystal growth behavior and the formation of structural defects.

Under the current energy and chemical synthesis scheme of shifting to a green and sustainable approach, developing efficient energy harvesting and chemical production methods using solar energy has been the focus of both industry and academia. Metal oxides are promising photocatalysts for environmental remediation and electronics due to the easy generation of charge carriers when stimulated with solar energy. They are cost-effective, environmentally friendly, and usually have high surface areas. Titanium dioxide (TiO_2_) is the most studied metal oxide photocatalyst for wastewater treatment and hydrogen production [16,17]. However, it’s only active under ultraviolet light. In comparison, ZnO as a semiconductor with a wide band gap, high thermal conductivity, and high electron mobility, has many advantages for photocatalytic applications compared to TiO_2_. As summarized in Table 1, compared to TiO_2_, ZnO has several advantages, including: higher electron mobility, better light absorption across a wider solar spectrum, potentially lower cost, and in some cases, improved photocatalytic activity for certain pollutants, especially when considering bacterial inactivation. ZnO has a broad direct band gap energy of ~3.37 eV. The large band gap of ZnO enables its better utilization of UV range light. Besides, ZnO also exhibits excellent antifouling and antimicrobial properties. It is anticipated that ZnO can also express better photocatalytic performance due to a higher electron mobility of ZnO (100−300 cm^2^/V·s) compared to that of TiO_2_ (0.1–4.0 cm^2^/V·s). Furthermore, the valence band (VB) position of ZnO is lower than that of TiO_2,_ which can generate hydroxyl radical with higher oxidation potential [18]. Therefore, based on the superior properties, ZnO is considered a promising alternative for TiO_2_. Some studies have shown that ZnO even has a higher photocatalytic efficiency than TiO_2_ in degrading hard-to-degrade organic compounds [19].

**Figure 3:**
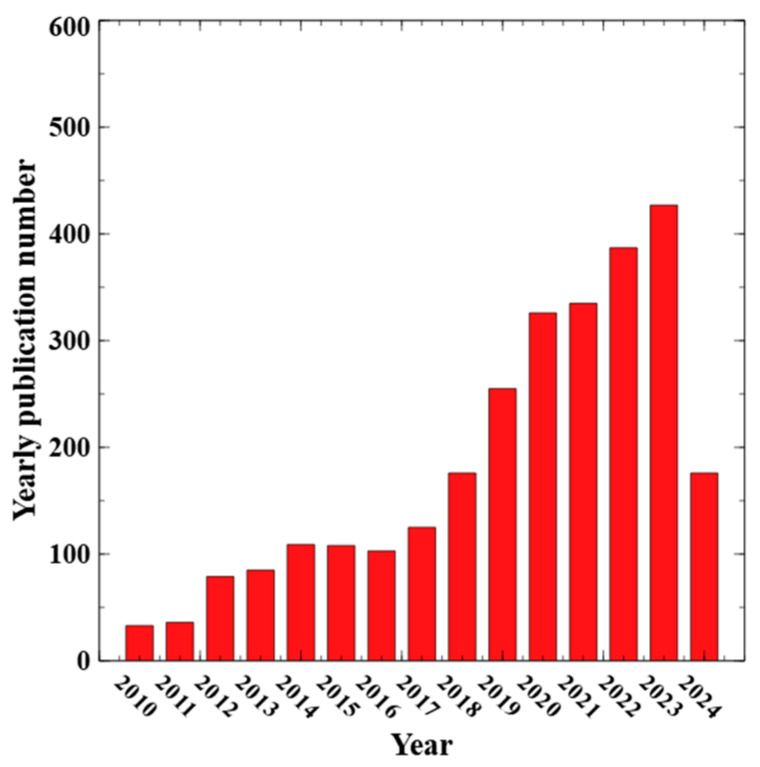
Number of publications per year for ZnO related photocatalysis from Web of Science search. Searching criteria: ZnO and photocatalysis*. Data extraction date: 8 May 2024.

Current research on ZnO has largely shifted toward the development of innovative nanostructures. Easily produced as a unipolar n-type semiconductor, ZnO with well-defined nanoscale morphologies and highly developed surface areas shows great potential for a wide range of applications. This is evident in the recent publication statistics, as shown in Figure 3. In terms of ZnO application in the realm of photocatalysis, publications have increased from less than 50 per year to ~450 in 2023 and the trend is still ongoing. The majority of the papers focus on ZnO nanomaterials dopant engineering, composite material formulation and structure engineering to increase the charge transfer efficiency, aiming to achieve higher solar energy utilization efficiency and catalyst activity.

Despite the extensive efforts and enduring interest on ZnO photocatalysis, its wide application and commercialization still suffer from many limitations, such as difficult catalyst-solvent separation, low light utilization efficiency due to fast electron-hole recombination, water corrosion, and limited light absorption etc. Many approaches have been conducted to tackle the challenges ranging from doping, defect engineering, surface engineering, heterojunction construction to composite formulation etc. Therefore, based on the fast-evolving photocatalytic applications of ZnO based materials, this review paper aims to provide a timely general summary of the current state of ZnO and its related materials’ nanostructures, synthesis strategies, working mechanism, and catalytic performance. Specifically, usage of ZnO related materials towards photocatalytic applications will be the main focus of the work, including modification and engineering approaches for achieving elevated ZnO photocatalytic performance. The literature search was conducted using keywords such as “ZnO”, “ZnO nanostructures”, and “photocatalytic applications”, as well as various combinations of these terms. References were gathered from reputable databases, including Web of Science, Google Scholar, and Scopus, to ensure reliability and academic credibility. Priority was given to studies published within the past ten years. At the end, current challenges and future directions in terms of ZnO photocatalysis will be discussed, aiming to serve as an introduction, a reference, and an inspiration for the coming research.

## 2. ZnO Nanostructure Synthesis

**Figure 4:**
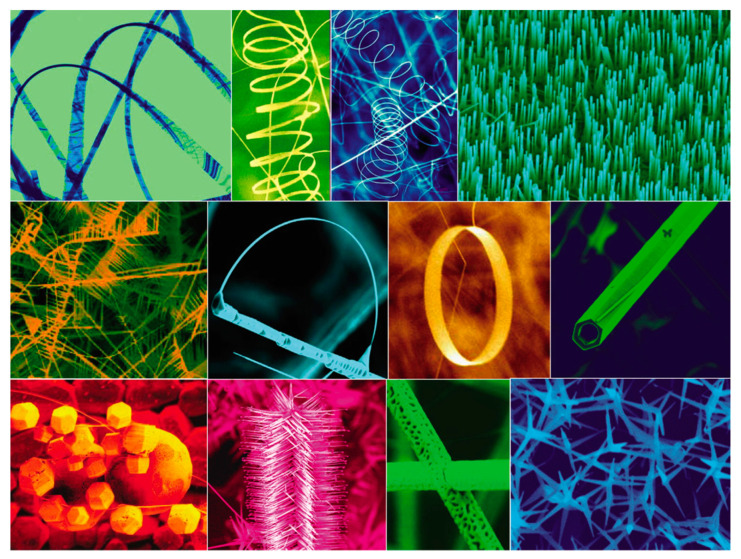
A collection of nanostructures of ZnO synthesized under controlled conditions by thermal evaporation of solid powders (from top left to bottom right: nanowire, nanohelix, nanorod, nanoweb, nanobows, nanoring, nanotube, nanocages, and nanopropellers of ZnO). Most of the structures presented can be produced with 100% purity. Adopted from ref. [22].

ZnO can be synthesized with a variety of morphologies, including nanoparticles, rods, wires, flakes, flowers, and sheets with semiconducting, piezoelectric, and pyroelectric properties. 1D ZnO structures include nano-rods, nanoneedles, nanohelixes, nanosprings, nanorings, nanoribbons, nanotubes, nanobelts, nanowires and nanocombs. Nanoplates or nanosheets represent the 2D forms of ZnO, whereas the 3D morphologies include structures such as flower-like shapes, dandelions, snowflakes, and coniferous or urchin-like formations. Novel nanostructures engineering and development has been a hot topic and is still the modern ZnO research trend due to the ease of fabrication using a wide array of methods with many possible shapes and sizes, offering tunable properties. In terms of ZnO nanomaterials synthesis, concentration, composition of the reagents, synthesis time, temperature, solution pH, and capping agents’ involvement all play important roles in determining the final ZnO nano morphology. According to the work of Vjaceslavs Gerbreders et al. [20], depending different synthesis parameters used, the ZnO morphology can vary from nano particle to nanoplates, nanowalls, and even hollow nanospheres. There are many review papers focusing on the ZnO nanostructure study. For nanostructure, thin film, epitaxial alignment and large ZnO crystal synthesis, reference [21] can be viewed with detailed summary and papers cited. For a detailed synthesis summary (methods, chemical, and synthesis conditions) of different ZnO nanomaterials, ref. [13] can be referred, with a specific focus on green synthesis approaches. The majority of studies on ZnO nanostructure formation rely on chemical synthesis methods. These typically involve reacting zinc salts, such as zinc acetate or zinc nitrate, with a base to form zinc hydroxide. This intermediate is then thermally dehydrated to produce ZnO and enhance its crystallinity. The reaction generally takes place in a solvent, and the resulting ZnO can be collected either as a powder or deposited onto a structured substrate. Besides, physical synthesis also offers a powerful tool for different ZnO nanostructures synthesis. A collection of nanostructures of ZnO can be synthesized under controlled conditions by thermal evaporation of solid powders, as shown in Figure 4.

**Figure 5:**
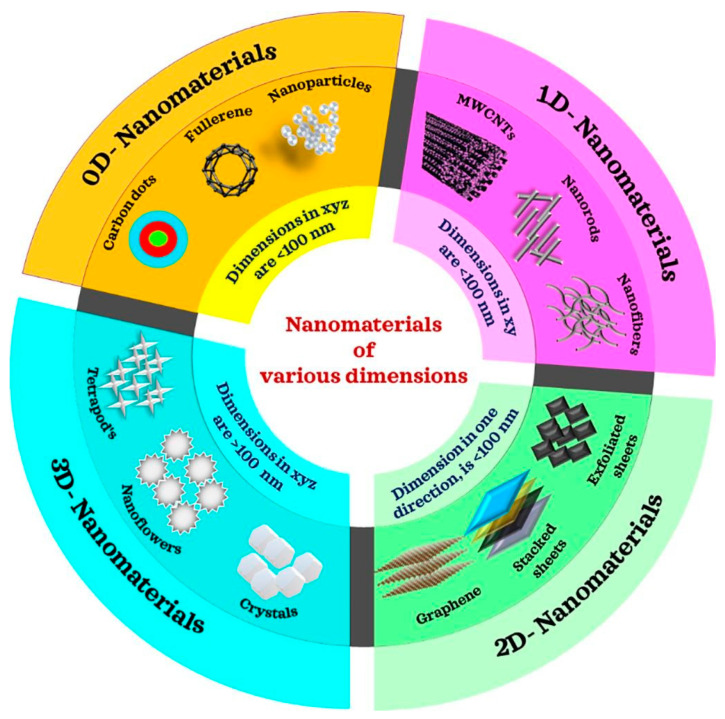
Graphical illustration, definition, and picture demonstration for nanomaterials of dimensions of 0D, 1D, 2D, and 3D. Adopted from ref. [23].

Up to date, ZnO nanostructure including nanospheres, nanoplates, nanorods, nanotubes, nanoneedles, nanoribbons, nanobelts, nanosheets, nanotrees, nanodendrites, nanoflowers, nanoshells, nanocorals, nanovolcanoes, nanopyramids, nanocolumns, nanotowers, nanocombs, nanorings, nanosprings, nanowires, nanocages, nanopencils, nano-pin-cushion cactus, and more have been successfully synthesized. In the work, a simple review on the ZnO nanostructure synthesis approaches for photocatalytic application will be presented with slight touch on their pros and cons. The nanostructures of ZnO are divided into 0D, 1D, 2D, and 3D in this review paper. An illustration of the different ZnO nanostructures can be viewed in Figure 5.

### 2.1. 0D Structure

0D ZnO structure represents nanoparticles (NPs). NPs are known as controlled or manipulated particles at the atomic level of 1–100 nm, showing size-related properties significantly different from bulk materials. Despite its simple morphology, ZnO NPs have been widely studied and applied in catalysis [21], electronics [24], rubber, food industry [25], agriculture [26], dentistry and antimicrobial area [25] etc. It also encompasses a high potential for modifications and properties tuning through surface and structure engineering. ZnO NPs synthesis can be achieved through various chemical and physical methods, including sol-gel [27], hydrothermal [28], co-precipitation [29], microwave synthesis [30], PVD, ball milling [31], and biological approaches [32] etc., each offering unique advantages for controlling particle size, shape, and properties. Since ZnO NPs have been extensively studied with many papers summarizing the synthesis methods in the literature. 0D ZnO will not be discussed in detail here. For a detailed synthesis methods and applications, the following review papers can provide ample information [26,33,34].

### 2.2. 1D Structure

1D ZnO nanostructures are characterized by two dimensions ranging from approximately 1 to 100 nanometers, with a significantly larger third dimension. These structures have attracted considerable attention in both academic research and industrial applications due to their potential as functional materials and as fundamental components for advanced systems. The literature commonly identifies four main types of 1D ZnO nanomaterials: nanotubes (NTs), nanowires (NWs), nanorods (NRs), and nanobelts (NBs) [35]. Owing to their physical dimensions, such NMs have exhibited novel optical, mechanical, and electronic properties. The basic mechanism of 1D ZnO structures’ fabrication is that ZnO seeding can have many growth surfaces with dynamic parameters, serving as a key for different 1D structure growth. Controllable preparation of 1D-ZnO can be achieved in many ways. Based on the growth mechanism, it can be divided into gas–liquid–solid (VLS) growth [36], gas–solid (VS) growth, dislocation-induced growth, and polar plane-induced growth. Based on the state of the preparation phases, they can be classified into the gas-phase method and the liquid-phase method [37,38].

Vapor deposition methods have been widely applied for ZnO 1D structure synthesis and include physical vapor deposition (PVD) and chemical vapor deposition (CVD). According to different synthesis methods and products, the source materials can be Zn, ZnO, carbon, or other powder-form materials. PVD produces vapor from raw materials through a physical process, without involving any chemical reactions during the phase or state transitions. The synthesis of 1D ZnO nanostructures using PVD involves two main steps: evaporation and deposition. Initially, ZnO powder is heated to high temperatures to generate ZnO vapor. This vapor then condenses and forms solid nanostructures, such as nanowires or nanobelts. Because ZnO has a high evaporation point of approximately 1975 °C, the PVD process typically operates at temperatures between 1300 and 1400 °C to effectively produce these nanostructures [37]. Compared to PVD, CVD involves both physical and chemical processes. In the method, Zn powder is commonly used as a starting material to generate Zn vapor at high temperatures. This vapor then reacts with oxygen to form ZnO. During the vapor-phase growth, controlling the growth kinetics allows for the formation of 1D ZnO structures with various morphologies. By carefully adjusting parameters such as reaction temperature, gas type and pressure, flow rate, deposition temperature, catalyst characteristics, substrate type, and positioning, a wide range of 1D ZnO nanostructures can be synthesized. In the CVD process, metal catalysts are usually used and play an important role for. In the work of Elisabetta Comini et al. [39], Au, Pt, Ag and Cu nanoparticles (NPs) were used to grow 1D ZnO with a VLS mechanism. They observed that morphology transition from nanowires to nanorods of ZnO can realized by using Au as catalyst with increased deposition temperature. While, ZnO (Au) nanowires and nanorods, ZnO (Pt) nanowires and ZnO (Cu) nanowires polycrystalline structure have preferred orientation for (002) growth. The morphology and surface difference finally result in a varied sensing performance for different gasses. Based on the VLS nanowire growth mechanism, positional, orientational, diameter, and density control of 1D ZnO nanowire can also be achieved [36].

The liquid phase reaction offers an easy 1D ZnO synthesis approach and includes liquid phase direct reaction, electrochemical deposition, and sol–gel methods. Solution-based synthesis methods can be performed at low temperatures (25–200 °C), making it compatible with many organic substrate materials and offering additional advantages such as straightforward processing, low cost, and ease of scale-up [38]. The liquid-phase direct reaction approach primarily includes the hydrothermal and solvothermal methods. The hydrothermal method involves reacting raw materials in an aqueous solution under controlled temperature and pressure conditions. One key advantage of solution-based synthesis is its ability to grow 1D ZnO structures on complex three-dimensional substrates, including those with pores and narrow spaces that vapor-phase methods may not effectively reach. Among these techniques, the hydrothermal method is the most widely used for producing 1D ZnO nanorod arrays on substrates.

Compared to liquid phase methods, advantages of vapor-phase 1D ZnO growth include highly crystalline ZnO nanostructures produced with minimal defects, which is essential for optoelectronic and sensor applications; controlled morphology (nanowires, nanorods, and nanotubes) by easy adjusting of growth parameters such as temperature, pressure, carrier gas flow, and catalyst presence; scalability for industrial applications; high aspect ratio 1D ZnO synthesized and versatility of substrate usage (e.g., silicon, sapphire, and glass). While there are many limiting factors, such as high temperature requirement, complex equipment system, limited growth control, slow growth rate, and potential catalyst contamination. For a solution-based approach, it offers a low temperature growth environment with cost effectiveness, high scalability, doping, and tunable possibilities for various large area and complicated substrates. Solution-based methods are particularly suitable for applications in sensors, photocatalysis, and bioelectronics where large-area deposition and chemical flexibility are advantageous. Therefore, vapor phase and solution phase synthesis approaches both provide valuable merits. Depending on different applications, vapor or solution-based methods need to be carefully considered.

### 2.3. 2D Structure

2D ZnO structures are materials where the ZnO is reduced to a sheet-like structure with a thickness of a few nanometers or less. 2D single crystals can be used as building blocks for 3D crystals preparation. 2D ZnO structures include nanosheets, nanoribbons, nanowalls, nanoflakes, nanoplates, nanodisks, nanoweb, and nanoring etc. [40]. Thanks to their high surface-to-volume ratio, 2D ZnO structures offer a large specific surface area and abundant active sites. When combined with their efficient charge transport capabilities and adjustable chemical and physical properties, 2D ZnO materials show strong potential for use in a wide range of applications, including batteries, supercapacitors, electronics and optoelectronics, photocatalysis, sensors, and piezoelectric devices [41]. Two primary strategies are used to synthesize 2D ZnO nanomaterials: the top–down and bottom–up approaches. Among these, the bottom–up method is more commonly used, as it tends to yield nanostructures with fewer defects and more uniform chemical composition. Top–down techniques include processes such as mechanical and liquid exfoliation, while bottom–up methods involve techniques like CVD and wet chemical synthesis.

Top-down methods for synthesizing 2D ZnO involve breaking down bulk ZnO material into smaller, 2D structures like nanosheets, and include techniques like mechanical grinding, laser ablation, and milling etc. For example, the liquid metal exfoliation technique has been used to produce ZnO nanosheets with lateral dimensions reaching the millimeter scale and thicknesses as thin as 5 nanometers. This method utilizes van der Waals forces between the interfacial oxide layer and the selected substrate to facilitate the exfoliation process [42]. A wet-chemical etching method is used for 2D spiral ZnO nanocrystals preparation from pyramid ZnO nanoparticles in a solvent mixture of oleic acid (OA) and 1-octylamine [43]. The formation of spiral structure is due to dislocations in the spiral shape surrounding the c-axis formed during the growth of the pyramid ZnO nanoparticles.

A bottom–up synthesis method includes chemical reactions that can be performed under optimized parameters and controlled precursor concentration. Strategies based on bottom–up synthesis are generally more adaptable to yield defect-free 2D ZnO nanosheets. A detailed summary of literature on 2D ZnO nanomaterials synthesis can be viewed in the previous reviews [41]. Using bottom-up approach, 2D ZnO nanosheets were grown by a solid vapor deposition process by Pingsun Qiu et al. at 1000 °C [44]. Reagent grade powders of ZnO, lead oxide, and graphite with molar ratio of 3:10:15 were mixed, ground, and placed in a tube furnace. They successfully grew a cluster of silk-like ZnO nanosheets onto a polycrystalline alumina substrate. The grown nanosheets are single crystals with a thickness of around 50–70 nm, a breadth of 50–100 μm, and length of 4–6 mm. The same method can also be used for preparing ZnO dendritic nanowires [45]. In the work, spontaneous formation of ZnO dendritic nanowires has been achieved on the faceted surfaces of polyhedral Zn microcrystals by oxidation at 600 °C. The Zn vapor was generated by evaporation of pure Zn metallic powder at 600 °C and transported by Ar gas. Then the synthesized sample was post annealed at 300–600 °C in an open tube at atmospheric condition. The authors found that higher temperature and longer oxidation time can lead to longer wires and an increased number of branches for the nanoweb structure. What is more, 2D ZnO nanosheets with 350–450 nm in transverse size and 80 nm in thickness are also synthesized and dispersed on both the surface and inside of the cellulose based thin films using a ZnCl_2_ precursor through a two–step hydrothermal synthesis method at room temperature [46].

### 2.4. 3D Structure

**Figure 6:**
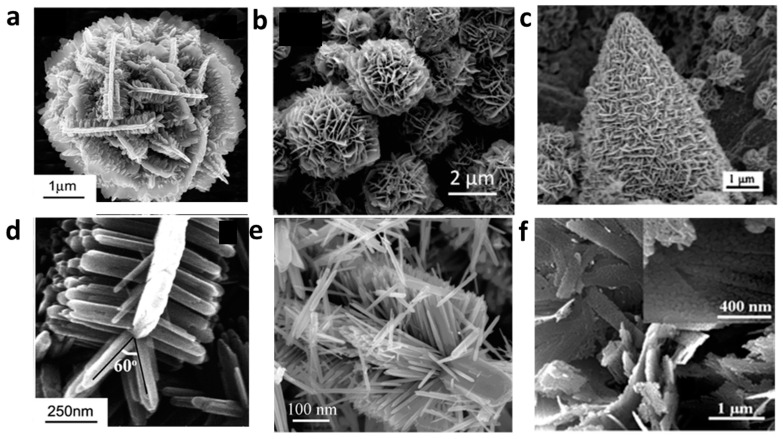
A SEM images collection of 3D hierarchical ZnO structure synthesized through concentration-controlled heterogeneous nucleation, solution-based self-assembly, solvothermal, sequential nucleation with hydrothermal synthesis, and zinc hydroxide carbonate calcination methods. (**a**) nanoflower [55], (**b**) nanoflower [56], (**c**) micropyramid/nanosheet [54], (**d**) nanofern [55], (**e**) nanobrush [57], and (**f**) porous nanosheet [58].

Three-dimensional (3D) ZnO structures are made up of nano building blocks that have been assembled into complex hierarchical structures through self-assembly. They have a number of unique properties, including: high surface area with a large surface area to volume ratio, porous structure, improved physical and chemical properties (better electron and ion transportation, increased reaction sites, and enhanced light harvesting, etc.). 3D ZnO structures have many applications, including: photocatalysis for hydrogen energy production, gas sensing [47], electrochemical sensors, and electrodes for lithium ion batteries [48] etc. So far, many synthesis strategies based on physical (physical and chemical vapor deposition, laser ablation, ball milling, lithographic, etc.), chemical (gas phase reaction, various solution phase synthesis), or biological methods have been well established to obtain 3D ZnO hierarchical nanostructures. Solution phase-based synthesis approach is still the main method used, including precipitation, microemulsions, hydrothermal/solvothermal, sol-gel, electrochemical deposition, chemical bath deposition, and so on [49]. 3D hierarchical ZnO nanostructures are commonly synthesized using the hydrothermal method combined with heat treatment. During the high-temperature process, the decomposition of precursors leads to the formation of porous structures. Additionally, oxygen vacancies can be introduced, with their concentration adjustable by controlling the calcination atmosphere. Depending on the desired structure or composite, various synthesis strategies may be applied, including multistep sequential growth, template-assisted methods, template-free self-assembly, and precursor or self-templating approaches [50]. A collection of 3D hierarchical ZnO structures synthesized through different methods can be viewed in Figure 6.

For instance, ZnO nanorod bundles were prepared through a simple hydrothermal reaction combined with calcination using Zn(Ac)_2_ (1.2 mmol) and urea (3.6 mmol) in a mixture of anhydrous methanol (25 mL) and deionized water (5 mL) by Lihua Huo et al. [47]. The bundle structure shows high gas response and selectivity for different drug analytes. Flower-like ZnO hierarchical nanostructures [51] with oxygen vacancies were prepared by using the hydrothermal method and calcination treatment in different atmospheres. The effect of calcination atmosphere on the oxygen vacancies and photocatalytic performance of ZnO hierarchical nanostructures play an important role. Hierarchical ZnO nanowires (ZNWs) and nanodisks (ZNDs) were also prepared through a two-step synthesis, including sequential nucleation and growth following a hydrothermal process by Ravi P. Silva et al. [52]. The hierarchical structure is assembled from initial ZnO nanostructures using a two-step seeded growth approach. It is learnt that the formation of these hierarchical structures depends significantly on the concentration of the growth precursor solution, growth time, and capping agent concentration. In another work, by using a template-assisted electrodeposition, a bottom-up epitaxial growth of 3D macroporous ZnO self-assembled nanostructures is coated on a p-type silicon substrate [53]. The ZnO formation is due to a chemical reaction between the Zn cations and the OH^−^ molecules that were present in the electrolyte. ZnO hexagonal micro-pyramid/nanosheet has also been prepared using a solvothermal method [54]. The 3D structure is composed of dense nanosheet-built networks that stand on a hexagonal-pyramid-like microcrystal. The hierarchical structure formation depends on the concentration of the ethylene diamine (EDA) solution as well as on the type of zinc source through a two-step sequential growth model.

**Figure 7:**
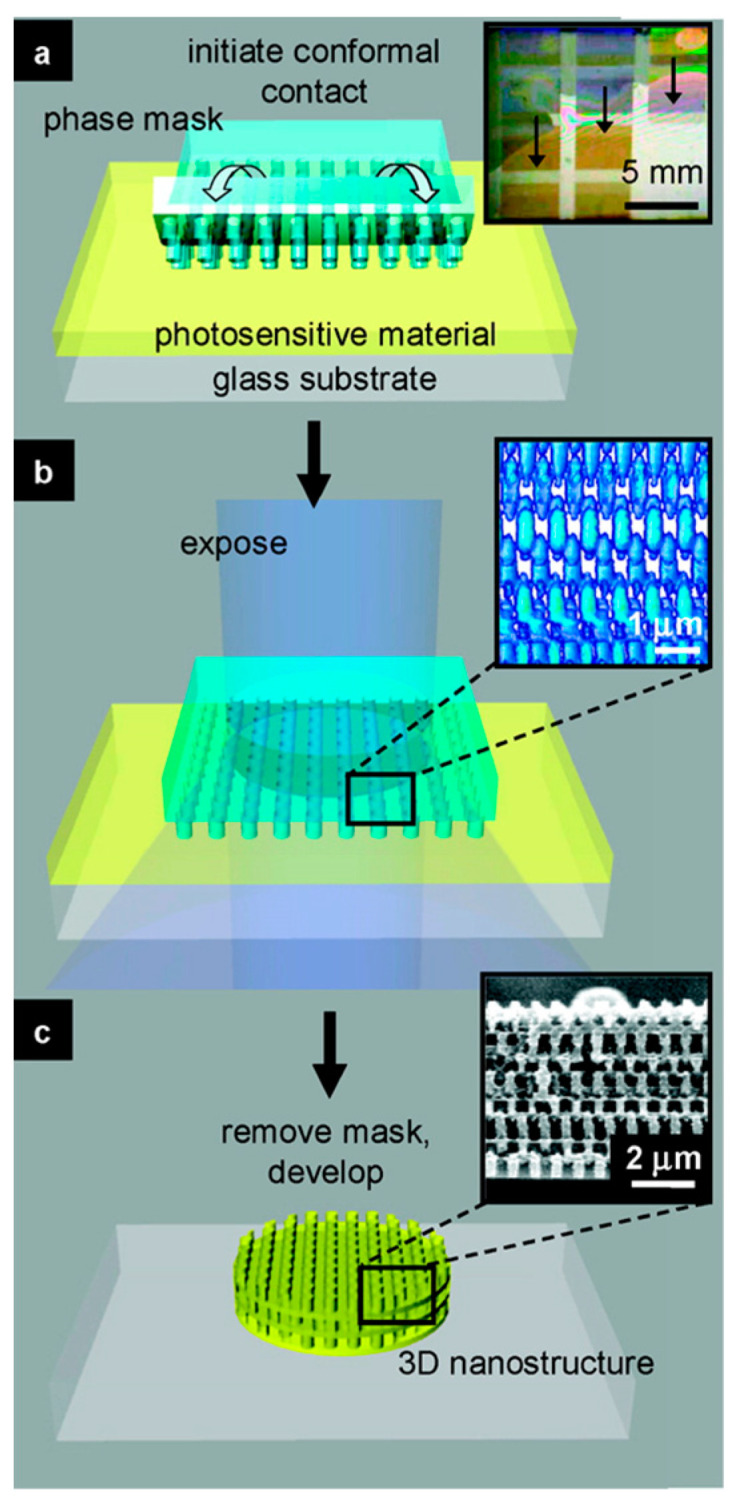
Schematic illustration of process steps for a proximity field nanopatterning (PnP) that uses high-resolution conformable, elastomeric phase masks to produce 3D nanostructures. (**a**) conformable mask coating, (**b**) light exposure, and (**c**) mask removal. Adopted from ref. [61].

Up to date, the most commonly used method of engineering diverse morphologies of 3D-ZnO superstructures is through the addition of a “growth modifier”. Modifiers play a crucial role in influencing the nucleation and growth rates of ZnO crystals, and can either promote or inhibit growth along specific directions, enabling face-controlled crystal development. Commonly used growth modifiers include surfactants, template agents, surface-directing agents, capping agents, and other chemical additives. These agents are essential for guiding the formation of complex hierarchies in 3D ZnO superstructures. For instance, surfactants, directing agent and template agent such as Ammonia, Trisodium citrate dihydrate, Triethylamine, Sodium dodecyl sulfate, Polygalacturonic acid, Cetrimonium bromide (CTAB), Heparin Glycol, Glycerol, *Cinnamon champora leaf*, and *Eryngium foetidum* L. can be used to produce 3D ZnO nanostructure as nanoforest, nanoflower, hexagonal ring, plate [59], multi-cage, donut-like [60], wool-ball, and spherical shape [49]. To be noted, under certain circumstance, several growth modifiers are necessary for either single or mixed forms. 3D ZnO structures can also be fabricated using other advanced methods, including: “proximity field nanopatterning” (PnP), a fabrication technique that relies on a conformable phase mask with sub-wavelength features of relief embossed onto its surface. The strategy is developed by Prof. John Rogers and Prof. Paul Braun at UIUC. The PnP process [61] includes three components: (i) a light source, which determines the wavelength, the intensity, and the angular and spectral bandwidth for the photo-exposure, (ii) a soft, elastomeric phase mask, which represents all of the necessary optics, and (iii) a photosensitive material capable of forming a solid structure in the geometry of the 3D distribution of intensity created by passing exposure light through the mask. A step by step PnP fabrication illustration is shown in Figure 7. In the work of Seokwoo Jeon et al. [62], they fabricated a 3D ZnO/ZIF-8 HNS as a highly sensitive and selective light-activated room temperature gas sensor. The 3D nanostructure fabricated using the PnP method shows an ordered and periodic nanostructure that offers effective gas diffusion and enhanced light absorption. There are a few literatures available using PnP method for 3D ZnO synthesis for photocatalysis applications mainly due to its drawbacks including potential alignment issues, limitations in pattern complexity, and the need for precise control over the gap between the mask and the substrate for optimal patterning quality. Other methods include robocasting technique (direct ink writing) [63], Micro-Patterned Lithography (MPL), and ACG can also be used to fabricate complex 3D ZnO nano structures. Despite the ability of creating complex 3D ZnO structures, those advanced methods are still in the R&D stage. Cost reduction and lack of easy scalability are the key issues for their wide applications.

In summary, there are many methods that can be used for 0D, 1D, 2D, and 3D ZnO structure synthesis. Even the same method can be tuned and altered to create different ZnO nanostructures. Therefore, it is important to choose a proper method based on the applications purpose. Currently, low temperature solution-based approach is the most widely used way of ZnO synthesis. By tuning the reaction chemicals and conditions, easy alternation of the ZnO nanostructure can be realized. For instance, Suib et al. [64] used zinc acetylacetonate as the zinc source with solvothermal syntheses method for varied ZnO structure synthesis. In the work, cauliflower-like, truncated hexagonal conical, tubular and rod-like, hourglass-like, nanorods, and spherical shapes were produced when THF, decane, water, toluene, ethanol, and acetone were used as the solvent, respectively. Fibers (1D), rhombic flakes (2D), and spheres (3D) can be prepared using the same sol-gel method with Zn(Ac)_2_ as the solute and various polyols as solvents (EG, G, and DEG) [27]. According to the authors, the morphology difference could be resulted from the chemical interactions between zinc ion and the solvents, forming different coordination configuration. A similar work by Motelica et al. also demonstrates the importance of solvents to ZnO nanoparticles’ synthesis [65]. Different alcohols were used for solvothermal synthesis, including a primary alcohol (methanol and 1-hexanol), secondary alcohol (2-propanol and 2-butanol), and tertiary alcohol (tert-butanol). ZnO nanoparticles can be successfully synthesized using primary alcohols, while no ZnO can be produced with the use of secondary or tertiary alcohols. Furthermore, depending on the alcohol used, ZnO with a structure ranging from quasi-spherical to rods can be obtained, with varied photocatalytic and antimicrobial activities. After a rough introduction of the synthesis methods for different ZnO structures. The following part will briefly discuss the mechanism of ZnO in the photocatalysis process including pollutants degradation, H_2_ generation, VOCs abatement, and CO_2_ reduction etc.

### 2.5. Category of ZnO Synthesis Methods

**Figure 8:**
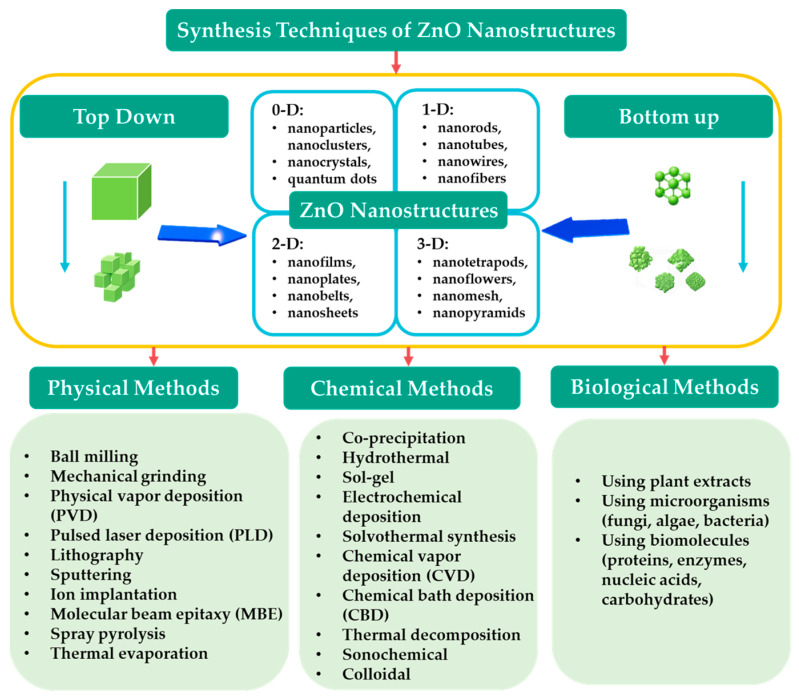
A schematic illustration summarizing ZnO nanomaterials structure and synthesis routes. Modified drawing from reference [40].

Zero-dimensional, one-dimensional, two-dimensional, and three-dimensional ZnO structures were covered in the previous sections. It seems that there are no fixed methods to produce a certain type of nanostructure. Instead, multiple nanostructures can be synthesized with the same method by changing the chemicals and parameters used in the synthesis. In conclusion, ZnO nanomaterials can be synthesized using a wide range of techniques, including solid-state reactions, coprecipitation, sol–gel processes, colloidal synthesis, chemical bath deposition, and various physical deposition methods, such as sputtering, vapor deposition (chemical or physical), and molecular beam epitaxy. The choice of synthesis technique and the resulting form of ZnO depends on the intended application. For instance, thin films are preferred for optical and electronic devices, while powders and colloids are more suitable for applications such as photocatalysis and biomedical uses. The form in which ZnO must be produced—either as a thin film deposited on a substrate or as a powder or colloidal suspension—can determine the method that should be applied. Usually, physical methods such as sputtering, PVD/CVD, and molecular beam epitaxy are used to produce high-purity ZnO thin films with well-defined structures. Powder or colloidal ZnO nanomaterials are typically synthesized using wet chemical and thermal methods that allow for precise control over the size, morphology, and dispersion of particles [66]. A summary of the ZnO nanostructures and their synthesis methods is shown in Figure 8.

Among the various methods for synthesizing ZnO, colloidal approaches to producing ZnO nanocrystals have attracted significant attention due to their versatility and the precise control they offer over particle size and morphology [67,68]. The method offers several advantages, including precise control, tunability, and surface functionalization, making it highly suitable for applications in photocatalysis, electronics, and biomedicine. Organometallic synthesis is a well-established method for producing colloidal ZnO nanocrystals with a controlled size, shape, and crystallinity [69]. The technique typically involves the use of organozinc precursors such as diethylzinc, zinc acetate, or zinc stearate, which undergo hydrolysis or thermal decomposition in the presence of suitable solvents and stabilizing ligands such as oleic acid, oleylamine, or trioctylphosphine [70]. During hydrolysis-based synthesis, water or alcohol is added to convert the precursor into zinc hydroxide, which subsequently transforms into ZnO. Under thermal decomposition, the precursor breaks down directly at elevated temperatures (typically 150–300 °C) to form ZnO nanocrystals. Stabilizing agents play a crucial role in preventing aggregation and directing crystal growth while reaction conditions, such as temperature, ligand concentration, and precursor ratio, are carefully adjusted to achieve a uniform ZnO morphology with high quality.

## 3. Mechanism of ZnO Photocatalysis

Photocatalysts are semiconductor materials that initiate and accelerate chemical reactions when exposed to light. In recent years, they have gained recognition as standard green catalysts due to their non-toxic nature and environmental compatibility. Upon exposure to light, an electron-hole pair can be generated within the semiconductor material. Notable examples of photocatalysts include TiO_2_ [71], ZnO [72], ferric (III) oxide (Fe_2_O_3_) [73], zirconia (ZrO_2_), vanadium oxide (V_2_O_5_), niobium pentoxide (Nb_2_O_5_), and tungsten trioxide (WO_3_), etc. Among the mentioned catalysts, TiO_2_ is the most widely used, followed by ZnO [74,75]. The working mechanism of all the photocatalysts follows the same rules. Photocatalysis begins with the use of light sources such as sunlight, ultraviolet (UV) radiation, or visible light, which provide the necessary photonic energy. When photons with energy equal to or greater than the band gap (BG) of the photocatalyst strike the material, they excite electrons (e^−^) from the valence band (VB) to the conduction band (CB), creating electron-hole (e^−^/h^+^) pairs. For the photocatalyst to function effectively, both oxidation reactions driven by h^+^ in the VB and reduction reactions driven by e^−^ in the CB must occur simultaneously [76].

**Figure 9:**
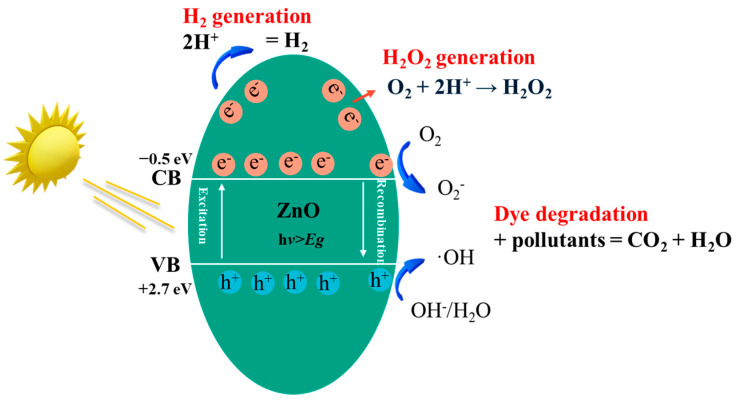
Schematic photocatalytic mechanism illustration of ZnO for various reactions, including H_2_ generation, H_2_O_2_ generation, and pollutants degradation.

In terms of the photocatalytic mechanism of ZnO for different applications, when ZnO is exposed to light with a photon energy greater than its band gap energy, electrons in the VB are transferred to the CB, creating e^−^/h^+^ pairs. The e^−^/h^+^ pairs then travel to the ZnO surface and participate in redox reactions. h^+^ combines with water and hydroxide ions to create hydroxyl radicals. While e^−^ combines with oxygen to create superoxide radical anions or H_2_O_2_. When the generated active species encounter organic pollutants, the organic pollutants are degraded into CO_2_ and H_2_O through a redox reaction. A detailed mechanism explanation for the photocatalytic oxidation steps involved with ZnO can be found in the work of [66,77]: (i) initially, the pollutants disseminate from the liquid phase to the outer surface of ZnO where adsorption takes place; (ii) During the adsorption process, redox reactions take place followed by desorption of the products, and (iii) Finally, the polluted products are removed from the interface. Figure 9 illustrates the detailed mechanism of ZnO photocatalysis. There are mainly three reactions illustrated using ZnO as a photocatalyst agent, namely H_2_ generation, H_2_O_2_ generation and pollutants degradation. Similar photocatalytic mechanisms are involved for the three types of reactions with the difference on reduction reactions where e^−^ goes. For H_2_O_2_, when exposed to light, ZnO acts as a photocatalyst, generating electron-hole pairs which can then react with dissolved O_2_ to produce H_2_O_2_ through a two-step process: initially forming superoxide radicals (O_2_^−^) and then further reducing them to H_2_O_2_ by gaining additional e^−^ [78]. In terms of H_2_ generation, in redox reactions at the surface of ZnO, H^+^ in water will be reduced by e^−^ to produce H_2_ gas, while the h^+^ oxidizes water to generate O_2_^−^ [79].

## 4. Photocatalytic Applications of ZnO

ZnO nanomaterials photocatalytic applications are mainly focused on environmental remediation, biomedical application and clean energy production [80], namely wastewater treatment, air purification, biomedical applications (antimicrobial, anti-cancer, and bioimaging), water splitting, hydrogen peroxide generation, and CO_2_ reduction etc. Detailed application for each will be discussed in the following parts.

### 4.1. Wastewater Treatment

Currently, ZnO used along is not sufficient for wastewater treatment. The primary challenges of using ZnO for photocatalytic wastewater treatment include its limited light absorption (primarily UV), rapid recombination of photogenerated electrons and holes, and potential for photo corrosion (leaching of Zn^2+^ ions), which can reduce its lifespan and introduce secondary pollutants. Therefore, most of the academic efforts are focused on ZnO modifications for improved photocatalytic efficiency and material stability. ZnO nanostructures and its composites have been extensively used for photocatalytic degradation of various pollutants, including organic dyes (like methylene blue (MB), methyl orange (MO), methyl red (MR), Rhodamine B, and Azo dye etc.), pharmaceutical drug (viz., anticancer, antidepressant, antibiotics etc.), heavy metal ion, ammonia and other contaminants in wastewater.

Organic dye photocatalytic degradation is the largest application arena for ZnO in the wastewater treatment with most of the papers published. For instance, modification of ZnO using precious metals is generally applied. High specific surface area and crystalline Ag−ZnO nanoparticle photocatalysts were synthesized by a one-step flame spray pyrolysis and tested for MB photodegradation [81]. The flame-made Ag−ZnO samples showed higher MB degradation efficiency than the wet-made ZnO and reference titania powders. The increased photocatalytic performance is due to the incorporation of Ag clusters acting to trap photo-induced electrons, retarding the electron–hole recombination process, and thereby, promoting the photocatalytic activity. Similar Ag–ZnO hybrid plasmonic nanostructures have also been prepared by a facile wet-chemical method using citrate as a directing agent [82]. Increased citrate concentration tended to form aggregated nanoparticles and a nano-disk shape at high concentration. The photocatalytic activity of Ag–ZnO hybrid nanostructures towards sun-light driven degradation of MB have been investigated and 94% of MB degradation can be achieved within 20 min duration much higher than 52% performance of bare ZnO due to the decoration of ZnO nanostructures with Ag nanoparticles, suppressing the recombination of photo-generated electrons and holes with improved sun-light utilization from plasmonic response of Ag nanoparticles. Ag–ZnO nanocomposites were also fabricated via a sol–gel route [83]. The photocatalytic performance of the catalyst was evaluated through the degradation of MB under xenon lamp irradiation. The results showed a significant enhancement in ZnO’s photocatalytic activity after modification with silver (Ag). Specifically, 1% Ag–ZnO achieved a 97.1% degradation rate of MB within just 15 min of light exposure. Using a scavenger test, the study confirmed that superoxide radicals (·O_2_^−^) are the primary reactive species driving the photodegradation process, and that Ag–ZnO heterojunctions facilitate the generation of more ·O_2_^−^ radicals. Additionally, a TiO_2_–ZnO (TZO) semiconductor–semiconductor (S–S) heterojunction nanoparticle with a low bandgap energy was successfully synthesized for enhanced photocatalytic applications [84]. The antibacterial activity of the materials was tested using *E. coli*, while photocatalytic degradation efficiency was assessed with both MB and MR dyes under direct sunlight. The TZO composite demonstrated strong antibacterial properties, producing an inhibition zone of 15 ± 0.8 mm against gram-negative bacteria, which was larger than that of ZnO nanoparticles (12 ± 0.2 mm). In terms of photocatalytic performance, TZO achieved approximately 25% degradation of MB and 13% degradation of MR under natural sunlight exposure. There are more organic pollutants with varied structure and chemical composition that can be degraded through ZnO photocatalytic degradation. More detailed information of the pollutants is summarized in Table 2. Many recent reviews have been published for ZnO’s applications for wastewater treatment. A recent review [85] on the photodegradation of organic pollutants using modified ZnO can also be referred. Specific review on drug degradation can be found in [86,87]. Tons of papers have been published using ZnO and its derivatives for organic pollutants photodegradation. However, limited commercialization has been demonstrated. Therefore, a detailed review paper summarizing the commercial challenges toward wastewater treatment is much needed.

Heavy metal ions stand for a huge group of wastewater pollutants and pose a great threat to human beings. Photocatalytic removal of heavy metal ions is a safe, green, and cost-effective approach and shows great promise. Heavy metal ions removal using ZnO particles synthesized through the solid precipitation technique has been conducted to study the removal mechanism [88]. The ability of ZnO particles to remove heavy metal ions—specifically Cu^2+^, Ag^+^, Pb^2+^, Cr^6+^, Mn^2+^, Cd^2+^, and Ni^2+^—from aqueous solutions was evaluated under both UV and visible light conditions. The results revealed that ZnO exhibited high removal efficiency (greater than 85%) for Cu^2+^, Ag^+^, and Pb^2+^ after one hour of UV light exposure. In contrast, significantly lower removal rates (less than 15%) were observed for Cr^6+^, Mn^2+^, Cd^2+^, and Ni^2+^. Based on various characterization techniques conducted before and after the removal tests, two primary mechanisms for metal ion removal by ZnO particles were identified: (i) physical adsorption and (ii) redox reactions facilitated by photo-generated electron–hole pairs. In summary, ZnO can remove heavy metal ions through one or both of these mechanisms, depending on the specific metal ions involved and the type of light used. In another work, Ag-tipped ZnO nanorod arrays decorated with glutathione-stabilized gold clusters (ZnO–Ag–Au nanorod arrays) have been successfully synthesized using a combination of photodeposition and electrostatic self-assembly techniques [89]. The photocatalytic reduction of Cr^6+^ using the prepared samples was tested under UV–visible light in the wavelength range of 300–800 nm. The ZnO–Ag–Au nanorod arrays achieved a 60% reduction of Cr^6+^ within 2 h, while maintaining their crystalline structure and Ag-tipped morphology. To better understand the photocatalytic mechanism, controlled experiments were conducted under varying light conditions. The results suggest that the formation of a semiconductor–metal/metal cluster heterostructure plays a key role, where Ag nanoparticles function as electron mediators and Au clusters act as visible light photosensitizers. This combination enhances charge carrier generation, promoting efficient charge separation and transfer, which contributes to the improved photocatalytic performance. Using a green synthesis approach, N–ZnO@Zeolite was prepared with efficient removal Cr^6+^ (93%) and Cd^2+^ (89%) under sunlight irradiation. The removal of the heavy metal ions includes adsorption and photocatalytic reduction. For a detailed summary of ZnO-based nanostorbents such as pristine ZnO NPs, doped ZnO nanostructures, ZnO nanocomposites, and surface-modified ZnO NPs along with the comparisons of their maximum adsorption capacity for different heavy metal ions (Cd^2+^, Hg^2+^, As^3+^, Pb^2+^, Cr^6+^, Ni^2+^, Co^2+^, and Cu^2+^), the review paper [90] can be referred.

Ammonia removal in wastewater using ZnO photocatalysts represents another great opportunity and extensive work have been conducted. Usually, ZnO along shows limited photocatalytic ammonia degradation efficiency. Composites are synthesized to increase the charge transfer, e^−^ and h^+^ generation. Chitin/ZnO was prepared via a sol-gel method to explore its photocatalytic activity for aquaculture wastewater treatment under UV irradiation [91]. Using orthogonal experiments to determine optimal conditions, the catalyst achieved 88.73% removal of NH_4_^+^ from synthetic wastewater with an initial concentration of 60 mg/L after 120 min of direct light exposure. Additionally, the results indicate that several operational factors—including mass ratio, catalyst dosage, calcination temperature, initial NH_4_^+^ concentration, and illumination conditions—significantly influence the efficiency of NH_4_^+^ removal. CuO/ZnO photocatalyst immobilized over the rough surface of a pottery plate was also prepared by an economical and easy method and used for water ammonia photodegradation [92]. In the optimum conditions, 77.2% ammonia removal from synthetic wastewater could be achieved at pH = 10.5. A two-step ZnO-modified strategy (Cu-doped ZnO nanoparticles, immobilized on reduced graphene oxide (rGO) sheets) for the promotion of photocatalytic degradation of NH_4_^+^ under visible light, was also reported [93]. Up to 83.1% of NH_4_^+^ (initial concentration 50 mg·L^−1^, catalyst dosage 2 g·L^−1^, pH 10) can be removed within 2 h under Xe lamp irradiation and the major by-product is N_2_. What is more, the composite shows a ~0.6% photocatalytic decrease after five successive trials. The catalyst is also tested under domestic wastewater conditions with simultaneous removal of chemical oxygen demand (COD), N, and P. The results show that the COD, total nitrogen (TN) and total phosphorus (TP) removal efficiencies can reach 84.3, 80.7, and 90.3%, respectively, indicating its potential commercial applications.

Besides the removal of each type of contaminants in wastewater in a single time, multiple contaminants removal at the same time can be achieved with proper materials design and is much preferred in practical working conditions. To achieve the goal, multi-functionality engineering on nanocomposites by combining 1D ZnO nanorods and 2D reduced graphene oxide (rGO) for efficient water remediation has been performed [94]. The nano-engineered ZnO NR–rGO nanocomposites showed efficient water remediation in terms of degradation of MB, MO, and RhB, and removal of Cu^2+^ and Co^2+^ heavy metal ions under visible light. The bifunctionality is a result of the high surface area and electron transport of ZnO and rGO combination. Similarly, a CuO–ZnO tetrapodal hybrid nanocomposite was prepared through a facile hydrothermal method [95]. The CuO/ZnO–T nanocomposite exhibited superior photocatalytic efficiency with 80% RY-145 dye removal and 86% BV-3 dye removal. In terms of heavy metal, 99% Cr^6+^ removal and 97% Pb^2+^ removal can be achieved, as compared to pristine ZnO-T under solar light. With various characterizations performed, the authors concluded that the dye removal is due to ZnO photocatalytic activity, while the heavy metal ions removal is due to adsorption. Other methods of improving the wastewater treatment efficiency are through construction of a hybrid system of photocatalyst materials with microorganisms [96]. The advantage of this approach lies in utilizing photocatalyst materials to harness free solar energy and generate high-quality photogenerated electrons, which help accelerate the rate-limiting step in microbial processes—specifically, the denitrification process. As a result, this method addresses the kinetic and efficiency limitations often seen in conventional treatment methods. The photocatalytic treatment of wastewater offers great potential, but it also highlights the need for the development of new, highly efficient photocatalysts. Despite extensive research, a comprehensive and effective solution for removing all types of organic pollutants from wastewater remains a challenge.

### 4.2. Air Purification

Volatile Organic Compounds (VOCs) have drawn significant research interest and emerged as a major public health concern. These compounds—such as aromatics, alcohols, ketones, alkanes, esters, and secondary pollutants like tropospheric ozone and peroxyacetyl nitrate—pose serious risks to human health. Various methods are available for VOC removal, including photocatalytic oxidation, adsorption, absorption, biofiltration, membrane filtration, incineration, and combustion. Among the methods, photocatalytic oxidation offers an effective way for sustainable, green, and large-scale approach for VOCs removal, especially for outdoor environment. ZnO nanoparticles, when exposed to UV light, can oxidize VOCs and other pollutants, converting them into less harmful substances like CO_2_ and H_2_O. For example, photocatalytic removal of 480 ppb NO under visible light over Cr-doped ZnO nanoparticles (Cr–ZnO NPs) has been performed by Viet Van Pham et al. [97]. In the study, Cr–ZnO NPs are synthesized by a sol–gel method with a narrow band gap. The results show that enhanced NO photocatalytic degradation performance (24.44% for 30 min under visible light), low NO_2_ conversion yield, and high stability under visible light could be achieved compared to plain ZnO. Ag/ZnO photocatalysts were prepared for effectively decomposition of ethyl acetate, toluene, and ethanol separately and in a mixed state. Due to significantly reduction of the photoelectron-hole pairs recombination in Ag/ZnO, 82%, 78%, and 73% of degradation efficiency, respectively, under visible light, can be achieved [98]. ZnO@Au core-shell structure with Au as core and ZnO as a thin shell was prepared for VOCs photocatalytic abatement [99]. The ZnO@Au showed excellent performance in the total oxidation of toluene (95%) and formaldehyde (85%) with the consequent formation of only CO_2_ and water as by-products due to efficient electronic communication between the gold core and the ZnO shell. A hierarchically structured Ag/ZnO/nBC sample based on biochar is developed for VOC photocatalysis with both mesopores and micropores, aiming to achieve a fast VOC capture and diffusion [100]. The Ag/ZnO/nBC can maximize the reactive oxygen species (e.g., ·OH and ·O^2−^) production and shows a 7.8 times higher degradation rate of formaldehyde than ZnO with high universality and stability. Chlorobenzene photocatalytic degradation is also demonstrated using metal doped ZnO nanoparticles (Ag/ZnO, Cd/ZnO and Pb/ZnO) [101]. About 100% of chlorobenzene removal efficiency can be obtained using Pb/ZnO nanoparticle within a short duration (<120 min) under visible light source, indicating the high potential of using modified ZnO as VOC photocatalyst. Work has also been conducted to increase ZnO photocorrosion resistance. Nanodiamond-decorated ZnO (ND-ZnO) catalysts have been prepared using a simple dehydration condensation process between taking advantage of the hydroxyl groups on the surface of ZnO and the oxygen-containing functional groups on ND [102]. Under UV light irradiation, nearly 100% degradation of toluene can be achieved within 120 min. Mechanistic studies reveal that both ZnO and ND can be excited under UV (365 nm) light to generate e^−^/h^+^ pairs. When ZnO is decorated with ND, the free electrons from ND transfer to ZnO, creating a built-in electric field. This field facilitates the transfer of h^+^ from ZnO to ND, allowing the oxidation of toluene to occur primarily on the ND surface rather than on ZnO itself. As a result, this process significantly reduces the photocorrosion of ZnO. All VOC degradation tests mentioned above were conducted in a batch system. A continuous plug flow photoreactor under UV irradiation of 254 nm was also applied for hexane elimination using ZnO impregnated into perlite granules [103]. Despite the low hexane to CO_2_ conversion (25%) and ZnO deactivation, the work provided valuable experience for realizing continuous VOCs photocatalytic abatement. The research on the VOCs photocatalytic degradation has been moving forward at a fast pace. The state-of-the-art of recently published papers on high-efficiency photocatalyst VOCs abatement can be viewed in [104]. Specifically, for carbon-based nanocomposites, the paper [105] can be referred.

A table summary for ZnO photocatalytic applications, including dye degradation, heavy metal removal, ammonia oxidation, and VOCs abatement, is shown in Table 2.

**Table 2:** A table summary of ZnO’s photocatalytic applications, including dye degradation, heavy metal removal, and VOCs’ abatement.

Year	Catalyst	Application	Synthesis Method	Dye	Concentration	Light Source	Conversion	Time Duration	Reference
2018	ZnO nanonuts	Photodegradation of paracetamol	Co-precipitation molecular imprinting	Paracetamol, methyl orange dye, and phenol	50 ppm	UV light	Paracetamol: 100% Phenol: 61%	180 min	[29]
2010	Dumbbell ZnO	Dye degradation	Microwave assisted hydrothermal	Methylene Blue	15 mg/L	365 nm light	99.60%	75 min	[30]
2019	ZnO nanosheet/Cellulose composite	Dye degradation	Hydrothermal	Methyl orange	20 mg/L	UV	100%	50 min	[46]
2018	Plat ZnO	Dye degradation	Chemical precipitation	Azo dye	10 mg/L	UV and solar light	UV: 95% Solar: 88%	UV: 240 min Solar: 80 min	[59]
2006	Ag-ZnO	Dye degradation	Flame spray pyrolysis	Methylene Blue	10 ppm	8 W UV tube	55%	60 min	[81]
2014	Ag-ZnO	Dye degradation	Wet chemical	Methylene Blue	10 μM	Sun light	94%	20 min	[82]
2021	Ag-ZnO	Dye degradation	Sol-gel	Methylene Blue	10 mg/L	UV	97.10%	15 min	[83]
2021	TiO_2_@ZnO heterojunction	Dye degradation	Template	Methylene Blue Methylene red	10 ppm	Sun light	MB: 25% MR: 13%	120 min	[84]
2017	ZnO nanoparticles	Dye degradation	Sol-gel, zinc acetate as precursor	Methyl orange	200 mg/L	UV light	99.70%	30 min	[106]
2009	Dumbbell ZnO	Dye degradation	Hydrothermal	Crystal Violet, Methyl Violet and Methylene Blue	15 mg/L	365 nm light	CV: 68.0% MV: 99.0% MB: 98.5%	75 min	[107]
2020	S-doped ZnO	Dye degradation	Hydrothermal	Rhodamine B and phenol	5 ppm	Visible light	Rhb: 100% Phenol: 53%	Rhb: 60 min Phenol: 180 min	[108]
2019	Fern ZnO	Dye degradation	Electrochemical deposition	Methylene blue, nitrophenol, and Rhodamine B	MB: 10 ppm NP: 10 ppm RhB: 5 ppm	UV light, natural UV-filtered sunlight	MB: 99.1% NP: 98.2% RhB: 97.1%	120 min	[109]
2018	Al doped ZnO	Dye degradation	Sol-gel	Indigo Carmine		Hg lamp	97%	180 min	[110]
2013	Al doped ZnO-AZO	Dye degradation	Combustion	Methyl orange	10 mg/L	Visible light and sunlight	99.50%	90 min	[111]
2013	ZnO_1−x_/graphene hybrid	Dye degradation	ZnO reduction and GO dispersion	Methylene Blue	10 ppm	UV and visible light	97%	300 min	[112]
2022	ZnO nanoparticles	Dye and antibiotic degradation	Hydrothermal	RR141, CR, and OFL	10 mg/L	Sunlight	RR141: 100% CR: 100% OFL: 97.1%	RR141: 20 min CR: 60 min OFL: 180 min	[113]
2020	SnO_2_/ZnO	Dye degradation	One step polyol method	Methylene Blue	na	UV	98%	30 min	[114]
2021	Green synthesized ZnO nanorod and nano particle	Dye degradation	Precipitation	Methylene Blue	10 ppm	Concentrated Sunlight	94%	120 min	[115]
2020	Green Au/ZnO	Dye degradation	Precipitation	Rhodamine B	10 ppm	UV	95%	180 min	[116]
2022	Hexagonal Plate like ZnO Particles	Dye degradation	Hydrothermal	Methylene Blue	1 × 10^−5^ M	UV	100%	60 min	[117]
2018	ZnO fine particle	Dye degradation	Flame spray pyrolysis	Amaranth Dye	10 ppm	Solar light	95.30%	75 min	[118]
2019	ZnO nanoparticle	Heavy metal removal	Solid precipitation	Cu^2+^, Ag^+^, Pb^2+^, Cr^6+^, Mn^2+^, Cd^2+^, Ni^2+^	50 ppm	UV and visible light	Cu^2+^, Ag^+^, Pb^2+^, Cr^6+^, Mn^2+^, Cd^2+^ > 85% Ni^2+^ < 15%	60 min	[88]
2018	Ternary ZnO-Ag-Au nanorod array	Reduction of aqueous heavy metal ions	Photodeposition method and electrostatic self-assembly	Cr solution	5 mg L^−1^	300 W Xe arc lamp	60%	120 min	[89]
2020	CuO/ZnO-T	Dye degradation Heavy metal removal	Hydrothermal	BV-3, RY-145 Cr^6+^ and Pb^2+^	40 ppm: dye 10–60 mg/L: heavy metal ion	Sun light	80% RY-145 86% BV-3 99% Cr^6+^ 97% Pb^2+^	30 min	[95]
2023	N-doped ZnO@Zeolite	Heavy metal removal	Dip-coating	Cr^6+^, Cd^2+^	10–100 mg/L	Sun light	93% Cr^6+^ 89% Cd^2+^	60 min	[119]
2016	CuO/ZnO	Photocatalytic oxidation	Mechnical mixing	As^3+^ solution	30 mg/L	UV light	94%	240 min	[120]
2016	CuO/ZnO-Pottery plate	Ammonia degradation	Dip-coating	Ammonia	85–510 mg/L	Visible/UV (280–390 nm)	77.20%	30 min	[92]
2018	Cu/ZnO/rGO	Ammonia degradation	Dip-coating	Ammonia	50 mg/L	Visible light	83.10%	120 min	[93]
2024	Ag/ZnO	VOC abatement	Photoreductionmethod	Ethanol, ethyl acetate, and toluene	--	Visible light	ethanol: 82% ethyl acetate: 78% toluene: 73%	--	[98]
2023	ZnO@Au core-shell	VOC abatement	--	Toluene, formaldehyde, ethanol	--	Visible light	formaldehyde: 85% toluene: 95%	--	[99]
2020	Ag/ZnO, Cd/ZnO and Pb/ZnO	VOC abatement	Solgel	Colorobenzene	20 µg/L	Fluorescent light, UV light, tungsten light and LED light	100% under visible light	120 min	[101]
2019	Nanodiamand-ZnO	VOC abatement	Dehydration condensation	Toluene	50 ppm in air	Xenon lamp	100.00%	120 min	[102]

### 4.3. Antimicrobial Application

**Figure 10:**
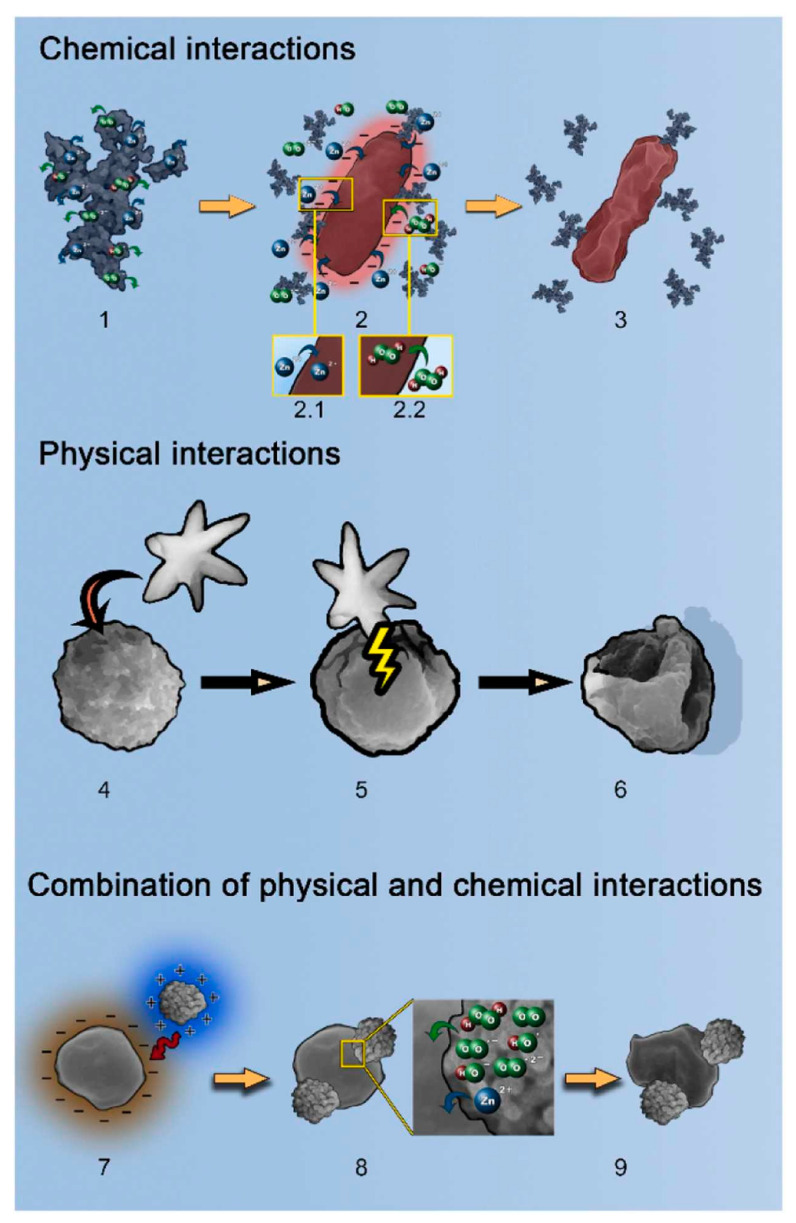
Multifaceted antimicrobial mechanisms of nanostructured ZnO: a comprehensive illustration (three types of mechanisms are shown: chemical, physical and a combination of chemical and physical approaches) [122].

Although synthetic chemicals and antibiotics have been effective in controlling and deactivating viruses and bacteria, their widespread use has led to increased drug resistance, further contributing to the spread of infectious diseases. As a result, there is an urgent need for alternative solutions that minimize adverse side effects. Semiconductor materials and emerging nanomaterials have opened new possibilities in photocatalysis and antibacterial applications. Among them, ZnO-based nanomaterials are widely regarded as promising antibacterial agents due to their strong photocatalytic activity, low toxicity, natural abundance, and the ability to tailor their properties for specific needs. Photocatalytic antimicrobial in water treatment using ZnO-based nanostructures has been extensively studied [121]. Besides, ZnO has also been integrated in construction materials (paint, mortar, concrete, wood, others). While the applications are still in the development stage and further investigation of the ZnO nanomaterials effect on the physical-chemical properties and durability of construction materials is needed [122]. The mechanisms of ZnO-based materials’ antimicrobial properties are summarized in Figure 10. The mechanism can be divided into non-contact and contact modes. Three main non-contact chemical antibacterial mechanisms exist: (1) generation of reactive oxygenated species (ROS), (2) release of Zn^2+^ ions, and (3) photoinduced production of H_2_O_2_. The chemical antibacterial mechanism is a non-contact mechanism, meaning that ZnO doesn’t need to directly touch the bacteria to exert its antimicrobial effect. Besides, ZnO nanoparticles can physically interact with bacterial cell membranes, causing membrane disruption and loss of cellular integrity, also known as the contact mechanism. The interaction can lead to leakage of intracellular contents and ultimately cell death. During most of the antimicrobial processes, chemical and physical mechanisms both present and work together to achieve the goal. For instance, PVP–ZnO NPs have been prepared via a co-precipitation method using polyvinylpyrrolidone (PVP) as a surfactant [123]. The ZnO NPs have a size of 22.13 nm with a spherical shape. The synthesized PVP–ZnO nanoparticles demonstrated strong photocatalytic performance, achieving 88% degradation of Reactive Red-141 azo dye with a 10 mg catalyst dose, and nearly 95% degradation with a 20 mg dose. Their antibacterial activity was also confirmed, showing inhibition zones of 24 mm against *Escherichia coli* and 20 mm against *Bacillus subtilis*. The study attributes the photocatalytic and antimicrobial effects primarily to the generation of ROS, as supported by a detailed 2,2-diphenyl-1-picrylhydrazyl (DPPH) free radical scavenging analysis. ZnO nano- and microparticles shapes and sizes also play an important role in cytotoxicity towards cancer cells and bacteria [124]. Different ZnO nano- and microparticles (nanoparticles—NP, nanorods—NR, hierarchical structures—HS, and tetrapods—TP) have been synthesized and tested for antibacterial activity against *Escherichia coli* and *Staphylococcus aureus*. The results showed that in all tested samples, bacterial reduction occurred in a concentration-dependent manner, and the specific surface area played a crucial role in determining antibacterial effectiveness. For ZnO nanoparticles (NPs), nanorods (NRs), and hollow spheres (HS), the primary antibacterial mechanism was the generation of ROS. Additionally, NRs and NPs exhibited direct interactions with bacterial cell walls and membranes, further enhancing their antibacterial activity. Although ZnO-based nanomaterials have shown great potential in organic pollutants removal and antibacterial properties in aqueous phase, there are still many challenges need to be further resolved, such as further understanding of long-term exposure toxicity for human with the nanocomposites, better visible light response and utilization, performance under real-life working conditions, and solubility and difficulty in recycling etc.

### 4.4. Other Applications

Besides the aforementioned applications, there are more challenging and complicated photocatalytic applications using ZnO-based nanomaterials, such as H_2_ generation, H_2_O_2_ production, and CO_2_ reduction etc.

Light-driven photocatalytic water splitting for H_2_ generation presents a promising route towards solar-to-chemical energy conversion, offering a possible solution for the current energy crisis. Rh/GaN–ZnO photocatalyst modified with Al_2_O_3_ has been prepared for water splitting reaction. The results demonstrate that the incorporation of ruthenium (Ru) significantly suppresses reverse reactions, leading to a substantial enhancement in overall photocatalytic water splitting performance. This improvement is reflected in the apparent quantum efficiency, which increased from 0.3% to 7.1% at 420 nm—representing more than a tenfold enhancement [125]. Water splitting for H_2_ generation using TiO_2_ nanoparticles (TiO_2_ NPs), TiO_2_ nanotubes (TiO_2_ NTs), ZnO nanowires (ZnO NWs) and ZnO@TiO_2_ core–shell structures has been studied under simulated sunlight [126]. The ZnO@TiO_2_ core–shell structures and TiO_2_ nanotubes (NTs) exhibit the highest photocurrent density and photo-conversion efficiency among the tested samples. The core–shell heterostructure achieves a photocurrent density of 0.63 mA cm^−2^ at 1.7 V_RHE and a maximum solar-to-hydrogen efficiency (STHE) of 0.073% at 0.9 V_RHE under AM1.5G sunlight illumination (100 mW cm^−2^). This enhanced performance is mainly attributed to the high electron mobility of the monocrystalline 1D ZnO core and the large specific surface area provided by the polycrystalline TiO_2_ shell. Doping of ZnO is an effective way of improving its photo response. P-type ZnO is especially the focus. Recent advances of p-type ZnO employed in photocatalytic water splitting can be found in the paper [127]. Despite many advancements in the area, the lack of rigor and reproducibility in the data collection and analysis of experimental results has hindered progress. Therefore, it is important to follow a robust protocol, proper characterization, and evaluation methods for overall water splitting. In particular, various pitfalls in photocatalysis research, best practices for reproducibility, and reliable methods for conducting rigorous experiments can be found in the review paper [128]. Besides, a timely summary and analysis of the past work are equally important to find out future directions and current challenges.

Hydrogen peroxide (H_2_O_2_) is widely applied in industrial processes including sterilization, wastewater treatment, fuel cells, and chemical synthesis due to tis effective, versatile and green oxidant characteristics. H_2_O_2_ is also regarded as a promising sustainable energy carrier with no harmful byproducts. H_2_O_2_ can be produced either by a direct two-electron O_2_ reduction process (O_2_ + 2H^+^ + 2e^−^ → H_2_O_2_) or by a consecutive single-electron O_2_ reduction route (O_2_ + e^−^ → ·O_2_^−^ followed by ·O_2_^−^ + e^−^ + 2H^+^ → H_2_O_2_). Photocatalytic H_2_O_2_ production, as a cheap and clean process, shows great promise. In this regard, ZnO-based materials can provide many merits, and extensive work has been performed. For instance, a novel covalent organic framework (COF)-based ZnO/TpPa-Cl composite is prepared by electrostatic self-assembly using 1,3,5-Triformylphloroglucinol (Tp) and 2-chloro-p-phenylenediamine (Pa-Cl) for H_2_O_2_ production under simulated solar light [129]. A maximum H_2_O_2_ evolution rate of 2443 μmol·g^−1^·h^−1^ under simulated solar light irradiation can be achieved. Similar work has been conducted using a ZnO/WO_3_ step-scheme [78]. The highest H_2_O_2_-production rate of 6788 μmol L^−1^ h^−1^ can be realized. The improved photocatalytic performance is mainly attributed to the formation of an interfacial internal electric field (IEF) within the S-scheme heterojunction, which promotes effective spatial separation of charge carriers. This allows electrons with strong reduction potential to actively contribute to H_2_O_2_ production. However, ZnO-based photocatalysts still face several challenges, including rapid recombination of electron–hole pairs, limited visible light absorption, and low product selectivity. Future research efforts should focus on enhancing efficiency, deepening the understanding of underlying mechanisms, improving selectivity, and advancing toward commercial applications. The strategies overview for improving the H_2_O_2_ production efficiency can be viewed in the review work [130], including a detailed table summary for reaction conditions and a reaction pathway summary of photocatalytic H_2_O_2_ production.

For CO_2_ applications, photocatalytic reduction of CO_2_ with H_2_O is most often applied for a clean process formulation, and the products are usually C1 species such as methane (CH_4_), methanol (CH_3_OH), carbon monoxide (CO), formic acid (HCOOH), and formaldehyde (HCHO). Various Zn-based materials have been applied for CO_2_ photocatalytic reduction. Zn-based M(salen)-COFs have been synthesized and tested for CO_2_ reduction to syngas under UV irradiation [131]. Among the synthesized materials, Co-TAPT-COF-1 displayed well-defined nanotube superstructures and demonstrated the highest activity for syngas production, achieving CO and H_2_ generation rates of 8.39 mmol g^−^¹ h^−^¹ and 11.31 mmol g^−^¹ h^−^¹, respectively. The study further revealed that by varying the metal species, coordination environments, and ligands within the M(salen)-COFs framework, the CO to H_2_ ratio in syngas could be effectively tuned to meet specific application needs. To improve the process efficiency, Ag-Cu_2_O/ZnO nanorods (NRs) hybrid photocatalysts are synthesized [132], and photocatalytic reduction of CO_2_ using H_2_O vapor is performed. The hybrid catalysts exhibit enhanced charge carrier separation and transfer, along with improved CO_2_ adsorption capacity, leading to significantly better performance in photocatalytic CO_2_ reduction to CO under UV–visible light compared to bare ZnO nanorods (NRs). Mechanistic analysis indicates that the presence of Cu_2_O increases CO_2_ chemisorption, while the formation of a Z-scheme heterojunction between Cu_2_O and ZnO promotes effective separation of photogenerated charge carriers. Additionally, incorporating Ag nanoparticles onto Cu_2_O further facilitates electron transfer due to the “electron sink” effect of Ag. This synergistic interaction—combining strong CO_2_ adsorption with efficient multi-step electron transfer—results in the notably enhanced photocatalytic activity of the Ag-Cu_2_O/ZnO nanorod system. Besides single products generation, a mixed product of CO, CH_3_OH, CH_4,_ and ethanol can also be produced using ZnO/g-C_3_N_4_ photocatalyst with CO as the dominant product [133]. As is known, producing products with more than one carbon for CO_2_ photocatalytic reduction is still challenging. The high photoactivity of ZnO/g-C_3_N_4_ photocatalyst is due to a synergetic effect between ZnO and g-C_3_N_4_. Besides experimental act, modeling work based on first-principles calculations is also conducted to exploit 2D ZnO sheets as a prototype system for CO_2_ photoreduction [134]. The results indicate that the 2D ZnO sheets possess not only suitable band edge positions and strong optical absorption, but also offer active sites for CO_2_ reduction. The selectivity of the reduction process is inherently influenced by the number of layers, while the catalytic activity shows a clear correlation with the position of the O 2p band center. Despite the fact that ZnO is a promising photocatalyst for CO_2_ reduction, its wide bandgap and visible light absorption limitations, along with challenges in product selectivity and reaction mechanism understanding, still hinder its practical applications. Besides, the reaction mechanisms still need to be further understood to guide future catalyst design. The review specifically focuses on the photocatalytic conversion of CO_2_ using ZnO semiconductor through the hydrothermal method is cited here [135].

### 4.5. ZnO Degradation Under UV Radiation (Photo-Corrosion)

Despite the effectiveness of ZnO as a photocatalyst under UV irradiation, it is susceptible to photodegradation through a process known as photo-corrosion, resulting in deteriorated performance over repeated cycles of operation and leading to decreased catalytic activity. This degradation is primarily caused by the photocatalytic activity of ZnO itself, which can lead to its own oxidation under light exposure. When ZnO is irradiated with UV light, e^−^ is excited from the valence band to the conduction band, leaving h^+^ in the valence band. These photogenerated holes can react not only with organic pollutants but also with the ZnO lattice, particularly surface Zn^2+^ ions, leading to oxidation and the subsequent release of Zn^2+^ into the solution, as follows: ZnO + 2h^+^ → Zn^2+^ + ½O_2_ [136]. This self-oxidation process results in structural damage, reducing the catalyst’s stability and reusability. Furthermore, the accumulation of photogenerated charge carriers without efficient separation accelerates recombination and intensifies the corrosion effect [137]. A recent work by M. Dimitropoulos et al. [138] studied ZnO photo-corrosion from a microstructure perspective under two controlled conditions, as follows: (i) the influence of light irradiation in the absence of pollutants, denoted as the internal mechanism, and (ii) under reactions taking place on the catalyst surface with pollutant molecules, referred to as the external mechanism. The authors noticed that, with prolonged UV irradiation up to 8 h, effective regeneration of the ZnO lattice can occur, recovering its photocatalytic activity to the starting point and providing new findings regarding the photo-corrosion process. To mitigate ZnO photodegradation, strategies such as surface passivation through plasma treatment [139], heterojunction formation [140], or noble metal modification [141] are often employed to suppress photo-corrosion and improve the photocatalyst’s long-term performance. 

### 4.6. Strategies for ZnO Photocatalytic Performance Enhancement

As mentioned in the previous sections, the photocatalytic applications of ZnO for different processes basically suffer from the same challenges. To enhance the photocatalytic performance of ZnO across different applications, four primary strategies can be applied:

1. Integrating a semiconductor with a lower band gap (Eg),
2. Introducing localized states either slightly above the valence band or just below the conduction band,
3. Creating color centers within the band gap, and
4. Modifying the surface structure of the material.

These improvements can be achieved through various techniques, including:

(i) Metal and non-metal doping;
(ii) Co-doping;
(iii) Constructing composite materials;
(iv) Atomic substitution;
(v) Sensitization;
(vi) Other advanced modification approaches [121,127,128,130,142].

For example, the properties of ZnO can be modified by introducing foreign elements through metal doping, non-metal doping, or by coupling it with other semiconductor materials. These modifications not only shift the light absorption range but also extend the lifetime of photogenerated charge carriers, enhancing overall photocatalytic efficiency. Additionally, structural defects, which can influence performance, may be introduced using simple ball-milling techniques, and some of these defects can be partially repaired through annealing treatment. The photocatalytic activity of the ZnO sharply decreased as the ball-milling speed and milling time increased [143]. Besides, the surface properties can be engineered by various synthesis methods and the addition of surfactant during the synthesis. For a detailed modification strategies summary, such as tailoring the intrinsic defects, surface modification with organic compounds, doping with foreign ions, noble metal deposition, heterostructure with other semiconductors and modification with carbon nanostructures in terms of photoactivity and stability improvement of ZnO, the review paper provides a good reference [144]. Due to the extensive literature available for ZnO-based photocatalytic applications, limited content is covered in this work. Therefore, a summary of the previously published review papers is listed in Table 3 for an easy topic lookup based on certain foci.

## 5. Discussion of Current Challenges and Future Opportunities

ZnO nanostructures have been widely researched and recognized as a promising photocatalyst for solar-driven applications, including the degradation of persistent organic pollutants, VOC removal, antimicrobial treatments, and chemical synthesis. This is largely due to ZnO’s low production cost—approximately 75% lower than that of TiO_2_ and Al_2_O_3_—its non-toxic nature, and its ability to absorb a broader portion of the solar spectrum compared to TiO_2_. Tons of literature have been published at a fast pace, focusing on the modification of ZnO with the introduction of doping, other metals, metal oxides or non-metals. The varied and wide photocatalytic applications of ZnO-related materials make a clear and thorough summary challenging. Despite many advancements in the lab conditions, there are few commercialization efforts. For instance, most of the work for organic pollutants photodegradation is conducted using simulated single or multiple compounds, far from the real-life working conditions. Many of the materials need incorporation of precious metals (Table 2), making the large-scale production questionable. Besides, ZnO in powder form is usually applied, making the recycling and recovery a problem.

There are mainly three technical challenges facing ZnO-related materials' photocatalytic applications, namely, the high recombination of electron-hole pairs, low visible light utilization, and poor product selectivity. Photogenerated electron-hole pairs can recombine quickly, which decreases the lifetime of active species. Besides, many materials are only active under ultraviolet light, which is expensive to produce with limited access. Interfacial charge recombination and long-term stability have collectively yielded poor electron injection efficiency and thereby low current density and efficiency of the ZnO based photocatalytic samples. What is more, poor control of the properties of individual building blocks and low device-to-device reproducibility are further areas that require further improvement. Currently, structural engineering, construction of heterogeneous structures, surface engineering, and doping are the main approaches used to tackle the challenges. However, the photocatalytic efficiency for certain applications is still not satisfactory. Therefore, future work can be focused on development of a photocatalyst with a wider light absorption; commercialization of ZnO based materials for environmental applications; green synthesis method with high sustainability and less environmental adverse effect, and mathematical models’ establishment for photocatalytic operations/systems in order to predict the quantum yield, kinetics and optimum conditions of the corresponding processes.

## Figures and Tables

**Table 1:** Properties summary between ZnO and TiO_2_.

Material	Bandgap	Carrier Mobility	Crystalline Structure	UV Adsorption	Growth Mode	Surface Activity	Stability
ZnO	Direct, 3.37 eV	100–300 cm^2^ V^−1^ s	Single crystalline	UVA absorption. Broad spectrum adsorption	Anisotropic	Mediate surface area	Easy for water corrosion
TiO_2_	Indirect 3.2 eV for anatase 3.0 eV for rutile	<1 cm^2^ V^−1^ s	Mainly in polycrystalline	UVB absorption	Isotropic	High surface area Ultra-high for anatase phase	Stable

**Table 3:** A table summary for the recent reviews published for ZnO photocatalytic-related topics.

Year	Title	Content	Reference
2022	92 years of zinc oxide: has been studied by the scientific community since the 1930s—An overview	Brief introduction of ZnO history, properties, and benefits, fabrication methods of ZnO, and prospective implementations of ZnO in many fields of industry.	[14]
2007	ZnO: Material, Physics and Applications	Summary of material growth, fundamental properties of ZnO and ZnO-based nanostructures and doping as well as present and future applications with emphasis on the electronic and optical properties including stimulated emission.	[145]
2022	ZnO nanostructured materials and their potential applications: progress, challenges and perspectives	Review of chemical methods of preparation of ZnO NPs. Green method for the synthesis of ZnO NPs. Modifications of ZnO with organic and inorganic compounds and multitudinous applications of ZnO NPs.	[13]
2019	ZnO as a Functional Material, a Review	Review of current state of ZnO structures and synthesis technologies, with the main development directions underlined as epitaxial, thin film, thick film or nanostructure.	[21]
2022	Recent Advances in ZnO-Based Nanostructures for the Photocatalytic Degradation of Hazardous, Non-Biodegradable Medicines	Review of comprehensive understanding of the degradation of antibiotics using ZnO-based nanomaterials (bare, doped, and composites) for effective treatment of wastewater containing antibiotics.	[77]
2023	A review on 2D-ZnO nanostructure based biosensors: from materials to devices	The review reports on the main advances in 2D-ZnO nanostructure-based biosensors, including syntheis method, biomolecule immobilization on ZnO nanostructure, and classification of ZnO biosensors.	[146]
2024	Photocatalytic activity enhancement of nanostructured metal-oxides photocatalyst: a review	The paper provides an in-depth analysis of the photocatalytic activity of nanostructured metal oxides, including the photocatalytic mechanism, factors affecting the photocatalytic efficiency, and approaches taken to boost the photocatalytic performance throughstructure or material modifications. The paper also highlights an overview of the recent applications and discusses the recent advancement of ZnO-based nanocomposite as a promising photocatalytic material for environmental remediation, energy conversion, and biomedical applications.	[147]
2023	Recent Advances in ZnO-Based Nanostructures for the photocatalytic degradation of hazardous, non-biodegradable medicines	The review presents and discusses recent advances in the photocatalytic degradation of widely used drugs by ZnO-based nanostructures, namely (i) antibiotics; (ii) antidepressants; (iii) contraceptives; and (iv) anti-inflammatories.	[77]
2022	A study on doping and Compound of zinc oxide photocatalysts	The paper summarizes the research on this aspect at home and abroad in recent years, introduces the doping of transition metal ions by ZnO, the compounding of ZnO with precious metals or other semiconductors.	[148]
2024	Current trends and future perspectives on ZnO-based materials for robust and stable solar fuel (H_2_) generation	The review examines ZnO-based photocatalytic H_2_ generation via water splitting with different modification strategies and explores future outlooks for improving its performance.	
2023	Preparations and applications of zinc oxide based photocatalytic materials	The review summarizes the preparation and application of ZnO-based composites with high catalytic performance, including modification strateties, applications, and future challenges.	[80]
2021	Photocatalysis by zinc oxide-based nanomaterials	In the book chapter, the authors have focused on the various techniques to modify the characteristics of ZnO and recent advancements in the synthetic strategy to develop highly efficient materials. The alteration strategies of properties of ZnO have been reviewed.	[142]
2021	ZnO Nanoadsorbents: A potent material for removal of heavy metal ions from wastewater	The review systematically summarizes the use of ZnO nanostructures for removing poisonous heavy metal ions from water. Various ZnO-based nanostorbents such as pristine ZnO NPs, doped ZnO nanostrutes, ZnO nanocomposites, and surface-modified ZnO NPs are reviewed thoroughly along with the comparisons of their maximum adsorption capacity for different heavy metal ions (Cd^2+^, Hg^2+^, As^3+^, Pb^2+^, Cr^6+^, Ni^2+^, Co^2+^, and Cu^2+^) in a tabular form.	[90]
2025	A review on modified ZnO to address environmental challenges through photocatalysis: Photodegradation of organic pollutants	The review summarizes the current research progress on ZnO-based nanomaterials specifically for photocatalytic organic contaminant degradation.	[85]
2022	Photocatalytic activity of zinc oxide for dye and drug degradation: A review	In this review paper, photocatalytic degradation of ZnO has been highlighted. ZnO anoparticles that have been investigated as photocatalysts for degradation of dyes, drugs and other pollutants are summarized.	[87]
2020	ZnO based nanomaterials for photocatalytic degradation of aqueous pharmaceutical waste solutions—A contemporary review	The review highlights the ongoing advancements to modify conventional ZnO into its advanced NCs counterpart as photocatalyst for degradation of PDs. A detailed mechanisms of common PDs degradation by ZnO based NCs have also been summarized.	[86]
2022	Current advancements on the fabrication, modification, and industrial application of zinc oxide as photocatalyst in the removal of organic and inorganic contaminants in aquatic systems	The review summarizes the recent advances in the fabrication, modification, and industrial application of ZnO photocatalyst based on the analysis of the latest studies from different aspects including ZnO based materials overview, modifications, applicability, industries use, and bio-inspired ZnO.	[149]
2022	Volatile organic compounds (VOCs) removal by photocatalysts: A review	This review tries to investigate the state-of-art of recently published papers on this subject with a focus on the high-efficiency photocatalyst.	[104]
2019	Integrated adsorption and photocatalytic degradation of volatile organic compounds (VOCs) using carbon-based nanocomposites: A critical review	This review provides a critical review of the related literature with focuses on: (1) the advantages and disadvantages of various carbon-based nanocomposites for the applications of VOC adsorption and photocatalytic degradation; (2) models and mechanisms of adsorptive-photocatalytic removal of VOCs according to the material properties; and (3) major factors controlling adsorption-photocatalysis processes of VOCs.	[105]
2022	Recent Progress in ZnO-Based Nanostructures for Photocatalytic Antimicrobial in Water Treatment: A Review	This review is a comprehensive overview of recent progress in the following concents: (i) preparation methods of ZnO-based nanomaterials and comparison between methods; (ii) types of nanomaterials for photocatalytic antibacterials in water treatment; (iii) methods for studying the antimicrobial activities and (iv) mechanisms of ZnO-based antibacterials. Besides, different doping strategies to enhance the photocatalytic antibacterial properties of ZnO-based materials, future research and practical applications are proposed.	[121]
2022	p-type ZnO for photocatalytic water splitting	In the Perspective, the authos discuss recent advances in the fabrication of p-type ZnO by different dopants and describe the benefits of p-type ZnO compared to n-type ZnO for photocatalytic applications. The difficulties and challenges of p-type ZnO employed in photocatalytic water splitting and future advancement of p-type ZnO in an emerging area have been also discussed.	[127]
2023	Photocatalytic H_2_O_2_ production Systems: Design strategies and environmental applications	This review article introduces the strategies for improving H_2_O_2_ production efficiency.	[130]

## Data Availability

No new data were created or analyzed in this study.
